# Feature extraction of 3D Chinese rose model based on color and shape features

**DOI:** 10.3389/fpls.2022.1042016

**Published:** 2022-11-29

**Authors:** Jin’fei Liu, Shu’li Mei, Tao Song, Hong’hao Liu

**Affiliations:** ^1^ College of Horticulture, China Agricultural University, Beijing, China; ^2^ College of Information and Electrical Engineering, China Agricultural University, Beijing, China; ^3^ College of Machinery and Architectural Engineering, TaiShan University, Taian, China

**Keywords:** Chinese rose flower, 3D model, feature extraction, classification method, shape distribution

## Abstract

Flower classification is of great importance to the research fields of plants, food, and medicine. Due to more abundant information on three-dimensional (3D) flower models than two-dimensional 2D images, it makes the 3D models more suitable for flower classification tasks. In this study, a feature extraction and classification method were proposed based on the 3D models of Chinese roses. Firstly, the shape distribution method was used to extract the sharpness and contour features of 3D flower models, and the color features were obtained from the Red-Green-Blue (RGB) color space. Then, the RF-OOB method was employed to rank the extracted flower features. A shape descriptor based on the unique attributes of Chinese roses was constructed, χ^2^ distance was adopted to measure the similarity between different Chinese roses. Experimental results show that the proposed method was effective for the retrieval and classification tasks of Chinese roses, and the average classification accuracy was approximately 87%, which can meet the basic retrieval requirements of 3D flower models. The proposed method promotes the classification of Chinese roses from 2D space to 3D space, which broadens the research method of flower classification.

## 1 Introduction

The Chinese rose is of great variety with significant differences in shape, such as peach, cone, egg, etc., and in color, such as red, blue, yellow, green, white, etc. It requires rich professional knowledge and personal experience to accurately classify them (Swetharani and Prasad, 2021). With the rapid development of computers and machine vision, the flower classification method based on 2D images has become a research hotspot. Anjani (Anjani et al., 2021) studied a convolutional neural network (CNN) algorithm for classifying rose flowers with RGB input images, which helps ordinary people with limited botanical knowledge. Sun (Sun et al., 2021) proposed a deep learning (DL) method for flower quality grading. The method transformed the 2D RGB image and the depth image of flower buds into fused RGBD information, the results proved the depth information is helpful for the classification of the maturing status. Taking disease leaves of Chinese rose as the research object, Yin (Yin et al., 2021) studied the feature extraction method from the disease region and proposed a flower disease classification model. Malik (Malik et al., 2022) analyzed the performance of traditional classification methods, and provided a machine learning-based identification approach of rose types by leveraging the features from their leaves. Tigistu (Tigistu and Abebe, 2021) studied a Chinese rose’s variety classification scheme using color and shape features, in which the color information was described by three statistical moments of the RGB, and the flower shape was described by Fourier coefficients. Wang (Wang et al., 2022) collected images of large-flowered chrysanthemum cultivars in two consecutive years and proposed a multi-information model based on deep learning to recognize and classify large-flowered chrysanthemums. Zhang (Zhang et al., 2021) employed Deep Convolutional Neural Networks (DCNN) to train a chrysanthemum variety recognition system, which converted the image into embedded space, and used the metric learning method to identify unknown chrysanthemum varieties. Wang (Wang et al., 2022) synthesized the pear flower images using the visual characteristics of the pear flower as input, and proposed an improved YOLOv4 model for precise detection of the pear flower in natural environment. Abbas (Abbas et al., 2022) collected ten kinds of flower images and constructed an optimized generalized DCNN for flower object detection, location, and classification. Liu (Liu et al., 2020) used computer vision and machine learning algorithms to classify seven kinds of commercial chrysanthemum tea. The 2D image only contains the flower information from one visual angle, and due to the inherent inter-class similarity and intra-class differences of flowers, the flowers that are shot from different angles may contain similar information. Besides, 2D images are vulnerable to natural conditions such as light, background, climate, and other factors. The above challenges make it tough to accurately classify flowers. Although the above researchers have made some achievements in flower classification, due to the limited information contained in 2D images, the classification accuracy is still not high enough.

The 3D models of flowers contain all domain information of flower morphology, which is an omnidirectional and multi-level information set of 2D images of flowers. With the increasing maturity of 3D modeling technology in the plant field, it is an inevitable trend to improve research methods from the traditional 2D space to the 3D space. Therefore, the 3D model could significantly improve the classification accuracy when identifying flowers with high similarity (Garbez et al., 2018; Ochiai et al., 2019; Dhar, 2019). The significant development in the fields of 3D modeling and digital archiving has led to an outburst in the amount of data stored digitally and model diversity (Manda et al., 2021). It is increasingly important to extract model features and realize their similarity retrieval quickly and effectively (Grabner et al., 2019). Retrieval methods of 3D models can be divided into statistical methods based on morphological features, view projection methods, and function transformation methods (Ruiz, 2021). Different methods have different emphases, but they all improve the retrieval effect of 3D models to varying degrees. Among them, the method based on color and shape distribution is widely used for its strong adaptability and wide application range (Zhu et al., 2022 ,Zhou et al., 2021). Kim (Kim et al., 2017) proposed a shape distribution-based 3D CAD assembly comparison method, which can enable a comprehensive comparison of 3D CAD assembly models. Curkovic (Ćurković et al., 2018) studied a re-distribution method of the matrix representation of the geometry and proposed a redistribution of the matrix representation of the 3D geometry based on shape features. Xie (Xie et al., 2016) proposed a novel 3D shape feature learning method to extract high-level shape features that are insensitive to geometric deformations of shapes. Wang (Wang et al., 2016) proposed a controllable anisotropic shape distribution method that can be applied to models with complicated geometry and topology. Bi (2022) successfully applied the shape distribution retrieval method to the analysis of the perspective distance-angle shape distribution of the garden landscape, which effectively improved the classification ability. Gonzalez-Barbosa (Gonzalez-Barbosa et al., 2022) used Kinect 2.0 to collect 3D point clouds of tomato seedlings to obtain their morphological characteristics to monitor the growth of tomato seedlings. Jayakumari (Jayakumari et al., 2021) designed the CropPointNet CNN model to segment crops semantically from a three-dimensional perspective, and applied high-resolution laser radar to classify cabbage, tomato, and eggplant based on objects. Zhang (Zhang et al., 2021) obtained the 3D point cloud of pomegranate trees through an RGB-D camera, then extracted and fused the shape and color features of the 3D point cloud to establish an SVM classifier. Jadhav (Jadhav et al., 2019) reconstructed the 3D model of fruit to obtain volume and color maturity features, and developed a non-destructive and accurate fruit grading system. Tsoulias (Tsoulias et al., 2020) applied LiDAR to scan apple trees and proposed an apple detection method based on reflectivity intensity and geometric features.

The morphological structure of the 3D Chinese rose model is complex and diverse, and the single form of shape features cannot comprehensively represent the Chinese roses. The feature points generated by the traditional shape distribution method are incompatible with the mesh area on the surface of the 3D model. The morphological changes of the 3D model structure are mainly represented by a small-area mesh. Therefore, the feature points extracted by the shape distribution method cannot accurately describe the detail of the Chinese roses. Aiming at applying feature extraction technology to 3D Chinese rose model, in this study, a new method based on rose color and shape features was proposed to improve the classification accuracy of Chinese rose species.

The contributions of this study are summarized as follows

(1) Inspired by 3D CAD model retrieval methods, the feature extraction method was successfully applied to the 3D Chinese rose models, which promotes the classification of Chinese roses from 2D space to 3D space.

(2) The proposed method combines color and shape features, which helps to meet the basic retrieval needs of 3D Chinese rose models. The constructed shape-feature descriptor for the unique attributes of Chinese rose flowers can be applied to various search applications to improve plant classification accuracy. Besides, the research results could improve the 3D data’s practicability of Chinese roses.

The rest of this study is organized as follows: First, the processing steps in the analysis and construction of the descriptor with color and shape features are described in section 2. In section 3, the experimental results are presented and discussed from different aspects. Finally, conclusions are given in section 4.

## 2 Materials and methods

The Chinese roses used in this study are kindly provided by the College of Horticulture, China Agricultural University. The experiment is carried out at the College of Horticulture, China Agricultural University. The specific experimental equipment and methods are described in the following sections.

### 2.1 Feature extraction of 3D Chinese rose model

#### 2.1.1 Shape-distribution characteristic

**Figure 1:**
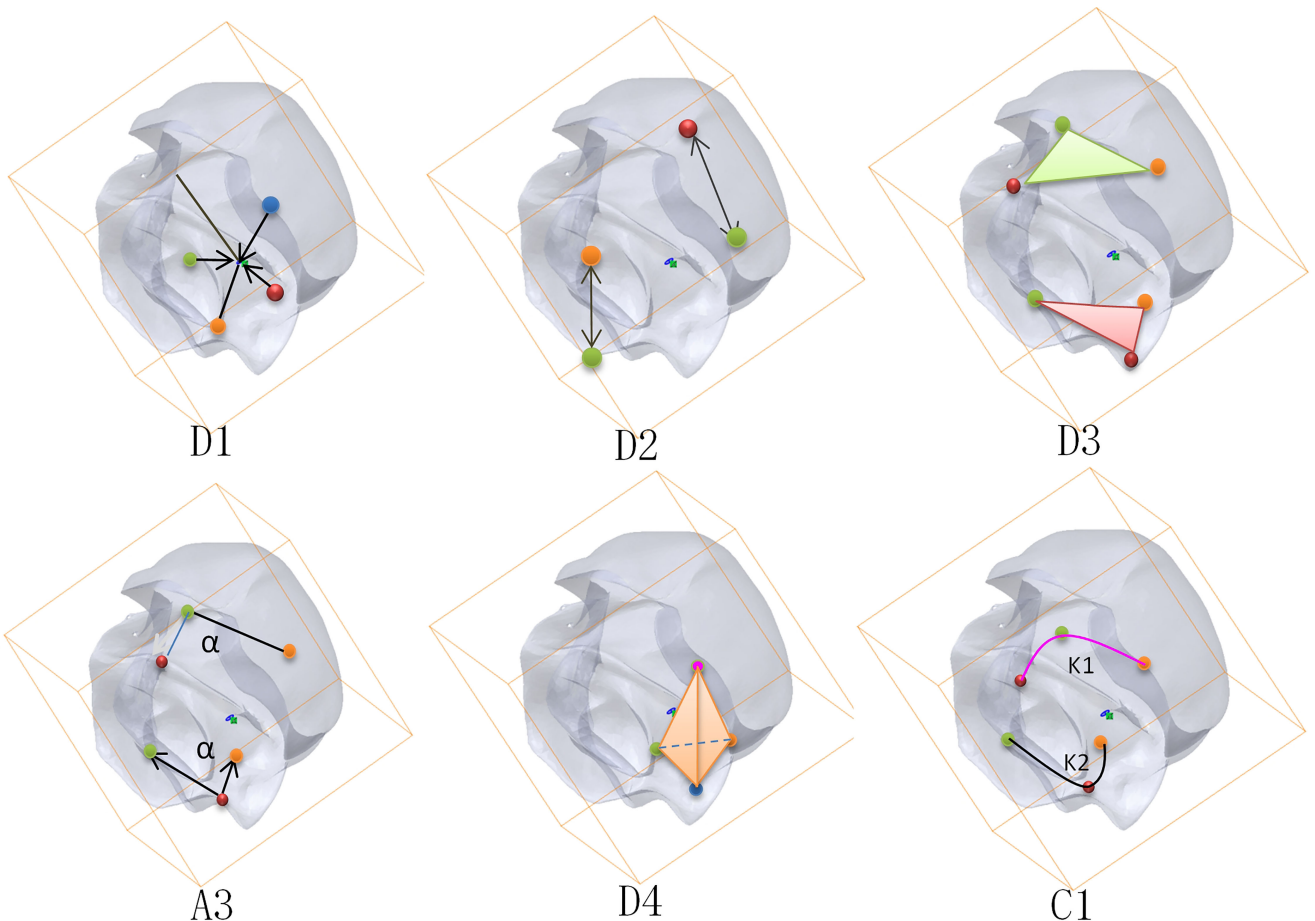
Characteristic diagram of common shape-distribution functions.

Shape distribution is one of the statistical probability methods, which takes distance, angle, length, and area of feature points as samples; it can transform the similarity measurement problem between complex 3D models into probability distribution matching (Liang et al., 2020; Liu et al., 2020). Among the 3D model feature functions, the model feature descriptor extracted by the shape feature function is the most effective. **Figure 1** shows the six commonly used shape distribution functions, and **Table 1** shows the attribute features of the corresponding shape distribution functions.

**Table 1:** Shape-distribution function properties.

Name	Feature	Number of points
Distance D1	Take the distance between the random point of the model surface feature and the center of gravity of the model as the sample	2
Distance D2	The distance between random points of model surface features was taken as the sample	2
Distance D3	The triangular area composed of random points on the surface of the model was taken as the sample	3
Angle A3	The angle between random points of model surface features was taken as the sample	3
Volume D4	The tetrahedral volume composed of random points on the surface of the model was taken as the sample	4
Curvature C1	The curvature of the circle composed of random points on the surface of the model was taken as the sample	3

The main factor affecting the feature extraction is the position’s direction. The rotation normalization caused by the change of position and size is the most complex. The six shape distribution functions shown in **Figure 1** are obtained by calculating the feature function between all the sampling points, and the six shape feature extraction of descriptors is rotation-invariant.

#### 2.1.2 Sharpness and contour features

The shape distribution feature is a general geometric feature; however, 3D models of plant flowers have specific morphological attributes. The attribute features of plant flowers were included to improve the effectiveness of the Chinese rose’s features. According to the extraction method of the 2D image, a special morphological feature descriptor for Chinese roses was constructed.

Sharpness is the quantitative indicator of plant visible defects, which can be used to describe the incomplete degree of the 3D Chinese rose models (Liu et al., 2020). Firstly, the center point of the 3D Chinese rose model was calculated, and its centroid was taken as the center point, which is defined as follows:


$$\left\{ \begin{array}{l} CenterX=\sum_{n=1}^{\text{sum}}x_{n}\\ CenterY=\sum_{n=1}^{\text{sum}}y_{n}\\ CenterZ=\sum_{n=1}^{\text{sum}}z_{n} \end{array}\right.$$

(1)

where the sum represents all mesh vertices of the 3D Chinese rose model; *x_n_*, *y_n_*, and *z_n_* represent the *X*, *Y*, and *Z* coordinates of the vertex of the *n*-th node.

Then, the Euclidean distance from the mesh vertex to the center point is defined as:


$$dis_{n}=\sqrt{{\left(x_{n}-CenterX\right)}^{2}+{\left(y_{n}-CenterY\right)}^{2}+{\left(z_{n}-CenterZ\right)}^{2}}$$

(2)

The ratio of the maximum to the minimum from the boundary point to the center point is then defined as the sharpness feature *S7* of 3D Chinese rose models:


$$S7=\frac{\max(dis_{n})}{\min(dis_{n})}$$

(3)

The sharpness feature represents flower fragility, while the contour feature represents the openness and fullness. Referring to the quantitative contour feature of 2D images, the 3D flower contour feature *P8* is defined as follows:


$$P8=\frac{V_{flower}}{V_{sphere}}$$

(4)

where *V_flower_* is the volume of 3D Chinese rose model; *V_sphere_* is the smallest volume of the ball that can surround the 3D Chinese rose model; the closer the *P*8 value is to 1, the fuller the rose flowers are.

#### 2.1.3 Color features

Chinese roses’ color can be divided into red, blue, yellow, green, white, etc. The corresponding relationship between rose color and species is shown in **Table 2**. A total of eight kinds of Chinese roses with different colors are considered in this study. Color information is complex and diverse, so it is critical to define the colors. In the process of 3D model reconstruction of Chinese roses, the 3D data of flower spatial location points were obtained, and the flower color information was captured at the same time. Therefore, the color space can be used to quantify the color features.

**Table 2:** Corresponding table of Chinese roses’ color and species.

Color	Rose flower	Color	Rose flower
Red	Hiogi, Movie star, Rouge Meilland, Baccarat, Kanegem, Fragrant Cloud	Blue	Blue moon, Blue bajou, Blue ribbon, Blue parfum, Blue river
Yellow	Golden scepter, Oregold, Pfalzer gold, Golden medallion, Princess michael of kent	Green	Green star, Green calyx
Pink	Pink fan, Pink peace, Guerlain, Duftrausch, Tang hong	White	BAIDUAN, Green cloud, Cygne antike, Tineke, White christmas
Black and red	Big purple, Black whirlwind, Norita Black pearl	Orange	Just Joey, Rosa.cv’KUNTELI,’ Rosa.cv ‘JINNIU,’ Nahema, Medallion

Commonly used color spaces include HSB, Lab, RGB, and CMYK. Each space represents a different range of color gamuts, and the specific calculation method is slightly different. Because the RGB color space is the most used and has a high matching degree in optical and physical equipment, it has the advantages of simple calculation and easy storage (Yuan et al., 2020). In the present work, the RGB space was selected to extract the color information of the Chinese rose as follows:

(1) Taking 50 color points randomly in the 3D Chinese rose model. Because green leaves could may interfere with the 3D reconstruction, the color difference *△E_n_* between *P_n_* (*R_n_*, *G_n_*, *B_n_*) and green RGB (191,212,143) is calculated as follows:


$$\Delta En=\sqrt{{\left(Rn-191\right)}^{2}+{\left(Gn-212\right)}^{2}+{\left(Bn-143\right)}^{2}}\;(1\leq n\leq 20)$$

(5)

If all *△E_n_* are less than the threshold *N*, the Chinese roses’ color indicator C9 = 6, which means that the rose flower is green. If the color difference of some sampling points is less than the threshold *N* and some is greater than the threshold N, it indicates that the color difference may be due to the interference of green leaves. Thus, the sampling points with color differences less than the threshold *N* will be eliminated, and the remaining color sampling points retained.

(2) Calculating the color difference between the selected sampling points of the eight kinds of Chinese roses separately. The RGB values and the color indicator of rose flowers with different colors are shown in **Table 3**. The color indicator of the minimum average color difference was the color of rose flowers, which finally determined the color feature of rose flowers: C9 (1≤C9 ≤ 8).

**Table 3:** Chinese roses’ color information.

Color	Value	Color	Value
Red (C9 = 1)	(217,32,37)	Blue (C9 = 5)	(49,130,237)
Yellow (C9 = 2)	(255,210,3)	Green (C9 = 6)	(191,212,143)
Pink (C9 = 3)	(245,163,199)	White (C9 = 7)	(204,205,200)
Black and red (C9 = 4)	(74,3,19)	Orange (C9 = 8)	(254,171,5)

### 2.2 Construction of fused feature descriptor

#### 2.2.1 Hierarchical sampling

Similarly, the Chinese rose’s centroid was taken as the center of the 3D Chinese rose model. After determining the model center, the Chinese rose’s center point was translated to the coordinate origin to realize translation normalization. Next, the maximum distance between each vertex on the flower surface and the flower centroid was determined, and the maximum radius scaling method was applied to process the model to complete the scale normalization of the mesh model.


$$F_{s}=\frac{F}{\underset{i}{\max}(dis(v_{i},o))}$$

(6)

where *F* represents the unnormalized rose model; *F_s_* is the normalized rose model; *dis*(*v_i_*,*V_m_*) is the distance between the flower surface vertex *v_i_* and center point *V_m_*.

**Figure 2:**
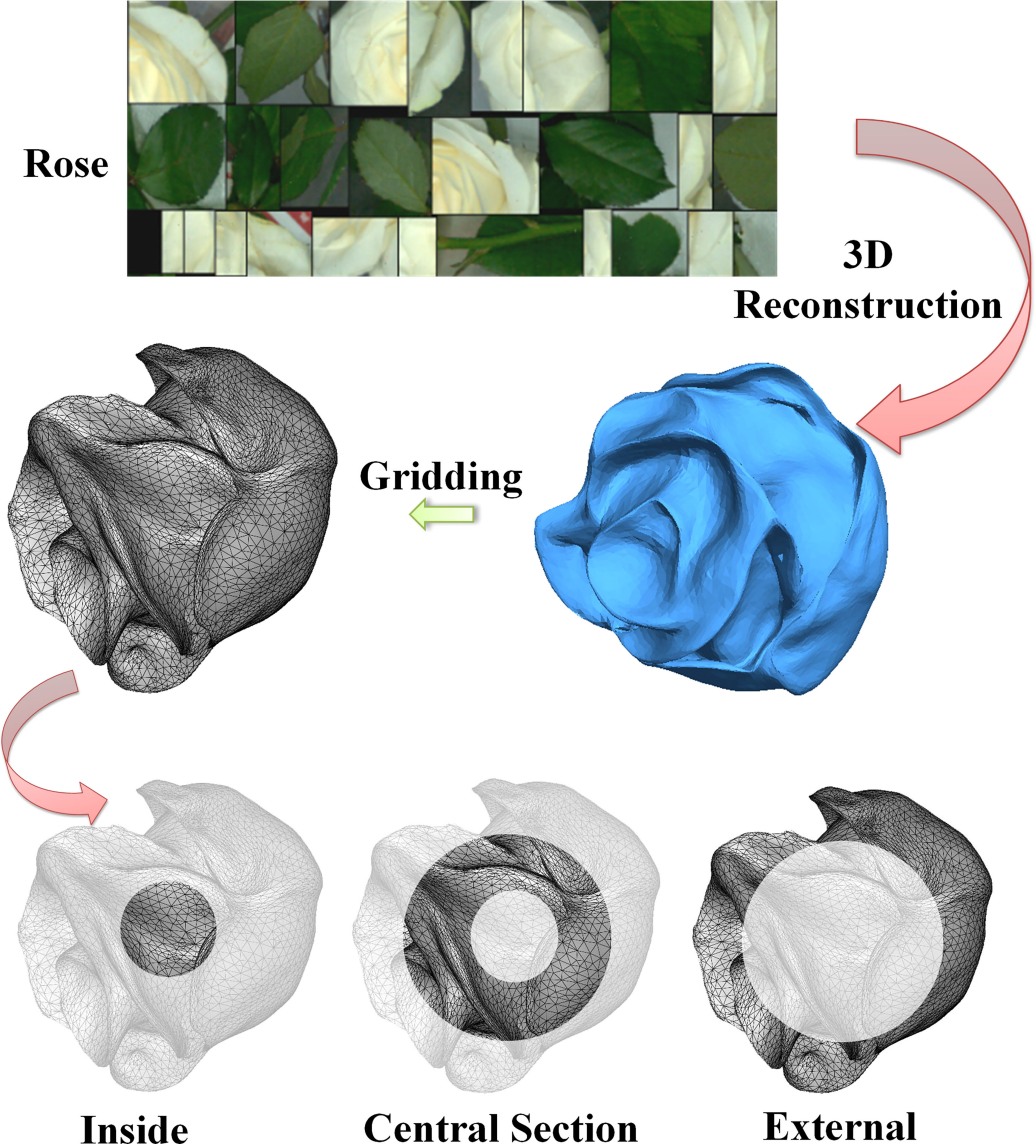
Stratification of the 3D Chinese rose model.

**Figure 2** shows the layering process of white rose flowers after the mesh. After normalization, the rose flowers were divided into three equal parts according to the volume of the sphere surrounded by the flowers. The distance between the sampling point and the center-line point was an ideal layering judgment indicator. Therefore, it was necessary to calculate the dividing point of the sphere diameter when the volume was divided into three equal parts.

According to the calculation formula of sphere volume, the sphere volume was divided into three parts. When the distance between the surface-point coordinates and the center point of the rose flower was (0.87,1), the flowers were divided into an outer layer (Out layer) representing the outer contour of petals. When the distance between the surface-point coordinates of rose flowers and the center point was (0.7,0.87), the flowers were divided into a middle layer (Mid layer) representing the internal morphology of petals. When the distance between them was (0,0.7), the flowers were divided into an inner layer (In layer) representing the stamen structure. The inner and middle layers represent the main form of the model, and the outer layer presents the detailed features of the model. The sampling points of shape distribution were crossed in turn, and points were taken from three different layers, which cannot only improve the efficiency of sampling points, but also fully extracts the inner, middle, and outer external structural features of 3D Chinese rose model, to characterize the structural attributes of rose flowers to the greatest extent.

#### 2.2.2 Feature fusion and similarity matching

The key to fuse the features is to determine the number and weights of features (Furuya and Ohbuchi, 2020). A total of nine features were obtained, including the shape-distribution features and the color features. In terms of weight, considering the diversity and complexity of Chinese roses, the RF-OOB method was selected to rank the importance of the nine features (Rahmana et al., 2022), and the feature weights were configured by gradient according to the order of importance (Yan et al., 2022).

The training dataset is composed of different kinds of Chinese roses with various colors. The nine features were combined in the order D1, D2, D3, A3, D4, C1, S7, P8, and C9. The error rate *e_t_* of features was tested without any weight when classifying rose flowers. The classification error rate $e_{t}^{j}$ was re-calculated after randomly replacing the *t^th^* feature in the sample. The difference between the error rate before and after the replacement was the important indicator of feature variables, the calculation formula is defined as follows:


$$V(X^{j})=\frac{1}{N}\sum_{t=1}^{N}\left(e_{t}^{j}-e_{t}\right)$$

(7)

RF-OOB is one of the ensemble learning approaches with high robustness; it can calculate the importance of output features completely, which has high applicability for rose feature extraction (Ruiz, 2021). Considering the retrieval efficiency, the distance measurement is the most appropriate for Chinese roses with a relatively simple 3D structure. Distance measurement can be understood as measuring the distance between points in different dimensions. The smaller the distance, the higher the similarity between different rose models. The types of distance measurement methods include Euclidean, Manhattan, and Hausdorf distances.

χ ^2^ distance is used herein to measure rose feature similarity.


$$d\chi^{2}(hist_{A},hist_{B})=\sum_{i=1}^{S}\frac{{\left(hist_{A}(i)-hist_{B}(i)\right)}^{2}}{hist_{A}(i)+hist_{B}(i)}$$

(8)

where *hist_A_* (*i*) and *hist_B_* (*i*) are the frequencies of the *i* ^th^ characteristic component of the *hist _A_* and *hist _B_* shape-distribution histograms of the rose models *F_A_* and *F_B_*, respectively; *S* is the sum of the number of histogram feature components and sharpness, contour, and color feature components.

The similarity calculation formula for the 3D Chinese rose model is:


$$Sim(F_{A},F_{B})=\frac{\max(Dis)-d\chi^{2}(hist_{A},hist_{B})}{\max(Dis)-\min(Dis)}$$

(9)

where max (*Dis*) is the maximum distance between the rose model *F_A_* and the rose model with the largest shape difference; min (*Dis*) is the minimum one.

## 3 Results and discussion

Fifteen kinds of Chinese roses were employed with 40 samples each. The variables of the shape distribution function are the number of sampling points and the number of distribution boxes. The number of distribution boxes reflects the resolution variable of the shape-distribution function. On the premise of ensuring the rapid classification of rose flowers, the sampling points of the shape-distribution function were set as 4000, and the number of distribution boxes suitable for the Chinese rose model was set as 400.

### 3.1 Component weight of fused features

The importance ranking of the 15 kinds of Chinese roses was performed by RF-OOB. The order of importance was C9, P8, C1, A3, S7, D1, D2, D4, and D3. Therefore, among all the characteristic components of rose flowers, color feature C9 is the most important one, and visual subjective information plays a leading role in the process of Chinese roses’ classification. And it can be seen that the contour feature P8, curvature shape feature C1, and angle shape feature A3 are at the top of the rankings; it can be perceived that the main contour, opening angle, and plumpness of flowers are the important characteristics of its shape, other features are the basic characteristics of flowers.

There are many ways to distribute weight values of characteristic features. The classification accuracy of average distribution weight and exponential distribution weight was measured. Considering the importance of color features and the diversity of rose flowers, the color-proportion feature Cp was added. The average distribution weight value is 0.1 and the exponential distribution weight values are C9 (0.28), Cp (0.19), P8 (0.14), C1 (0.11), A3 (0.08), S7 (0.07), D1 (0.05), D2 (0.04), D4 (0.02), and D3 (0.02). The experimental results with exponential distribution weight method and average distribution weight method are shown in **Table 4**.

**Table 4:** Classification accuracy of 3D Chinese rose models.

Number	Name	Classification accuracy of average distribution weight method	Classification accuracy of exponential distribution weight method
1	Golden Phoenix	76%	85%
2	Lady Figaro	92%	93%
3	Scentimental	81%	82%
4	Venus	94%	94%
5	Red Dragon	85%	88%
6	Double powder	91%	92%
7	Generation beauties	88%	88%
8	Guerlain	90%	91%
9	Soeur emmanuelle	83%	89%
10	Julio	90%	91%
11	Swan antique	93%	93%
12	Scarlet queen	82%	86%
13	Red Da Vinci	87%	92%
14	Golden Jade	71%	86%
15	Star	96%	96%

It can be seen that the classification accuracy of the average distribution weight method is greater than 70%, the average classification accuracy is 87%, the lowest classification accuracy of the exponential distribution weight method is 82%, and its average classification accuracy is 90%. The average classification accuracy of the exponential distribution weight method is 3% higher than that of the average distribution weight method, which reflects the advantages of RF-OOB, indicating that the main features should be highlighted in the process of Chinese roses’ feature classification; it is necessary to expand the weight of main feature components. Further comparative analysis shows that, on the premise of the high classification accuracy of the average weight method, the improvement of the exponential distribution weight method is not obvious, and the improvement amount is approximately 2%, while in rose categories with a low classification accuracy of average weight, the improvement effect of the index distribution weight method is more significant, with an increase of approximately 7%. This means that when the color and shape of rose flowers are greatly different, the exponential weight method basically does not work, while when the shape of rose flowers is relatively similar, the advantage of the exponential weighting method is more prominent. RF-OOB combined with the exponential distribution weight method is more suitable for 3D Chinese rose models. The morphology of the Golden Phoenix species is similar to that of the Golden Jade species, resulting in a low classification accuracy, while the classification accuracy of Lady Figaro, Star, Venus, and other species is high due to their particularity.

### 3.2 Retrieval tests of Chinese roses

**Figure 3:**
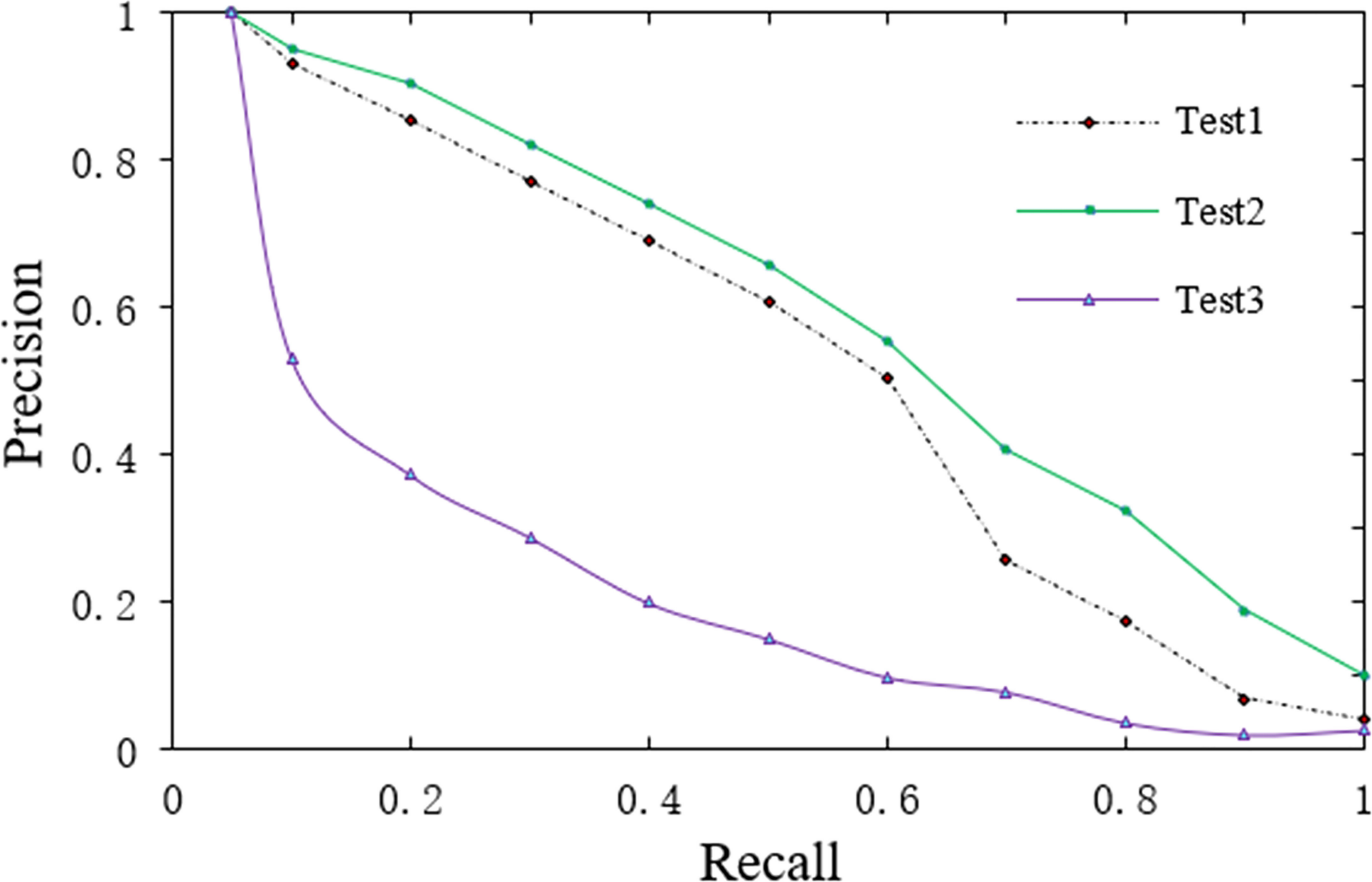
Precision-recall curve of Chinese roses’ retrieval.

Precision ratio and recall ratio were applied to verify further the proposed shape feature extraction and retrieval method for 3D Chinese rose models. They are essential indicators representing the effectiveness of information retrieval. In this study, precision ratio means the accuracy of the same kind and similar shape of rose flowers in the samples, while recall ratio means the comprehensiveness of the same kind and similar shape of rose flowers in the samples. The two indicators verify the accuracy and comprehensiveness of retrieving rose flowers based on 3D Chinese rose models. Generally, the retrieval test is conducted with a graph with the recall ratio as the abscissa and the precision ratio as the ordinate. The ideal precision-recall curve should be a line graph with an area of 1. **Figure 3** shows the retrieval results of three methods for testing rose flowers. The test 1 curve is the retrieval curve of the fusion feature of RF-OOB-1 (adopting the color feature and average distribution weight method), the test 2 curve represents the retrieval curve of RF-OOB-2 (adopting the color feature and exponential distribution weight method), and the test 3 curve represents the retrieval curve of RF-OOB-3 (excluding the color feature).

As seen from **Figure 3**, under the same recall ratio, the precision ratio of test 3 is significantly lower than that of test 1 and test 2. Compared with test 1 and test 2, the largest difference in test 3 is about the color feature. Therefore, it is inferred and analyzed that the color feature is a necessary and sufficient feature for the classification and retrieval of 3D Chinese rose models. Connecting the upper left-hand corner to the lower right-hand corner of **Figure 3**, it is found that the curves of test 1 and test 2 are basically on the upper diagonal, indicating that test 1 and test 2 can basically meet the needs of classification and retrieval. Further comparison shows that when the recall ratios of test 1 and test 2 are less than 0.6, the precision ratios are similar, while when the recall ratio further increases, the result of test 2 is significantly better than that of test 1. The difference between test 1 and test 2 is the difference in the distribution method of feature component weight when fusing features. This indicates that when the recall ratio is low, the exponential and average weight methods have little impact on retrieval accuracy, while when the requirements of retrieval comprehensiveness are high, the advantages of the exponential weight method are highlighted.

### 3.3 Case study

**Figure 4:**
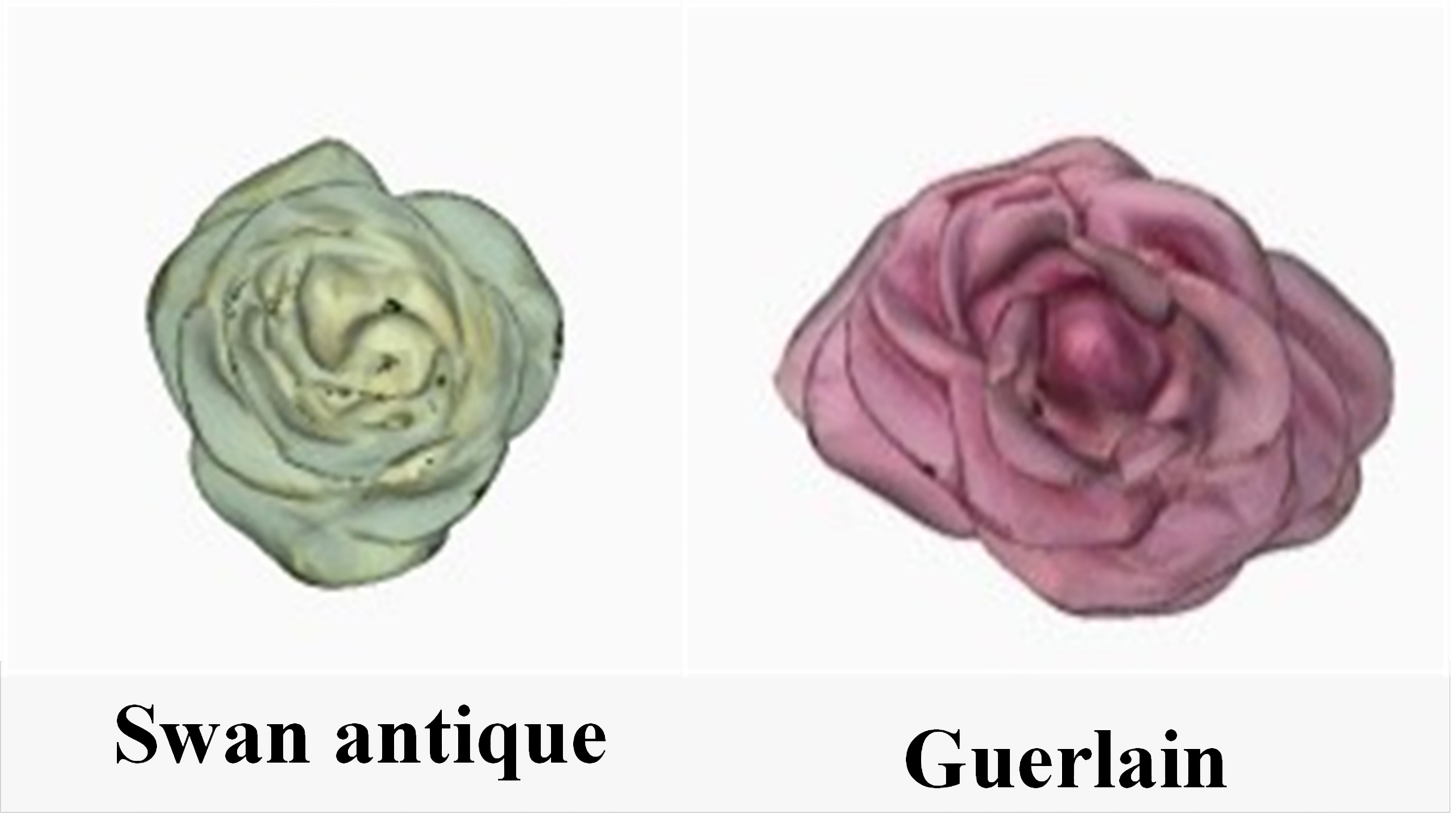
Input 3D models of Chinese roses.

**Figure 4** shows the input 3D models of Swan antiques and Guerlain roses. **Tables 5**, **6** show the similarity retrieval results of Swan antiques and Guerlain roses, respectively. A total of six related models were returned in the search results.

**Table 5:** Retrieval results of 3D models of Swan antique rose flowers.

Method		Retrieval similarity					
D2		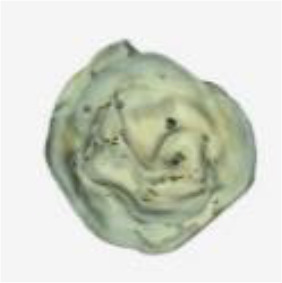	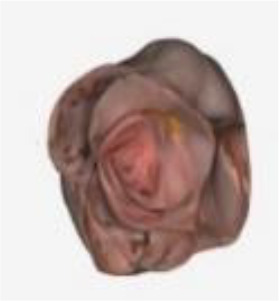	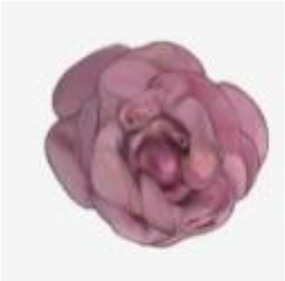	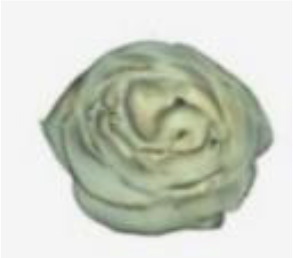	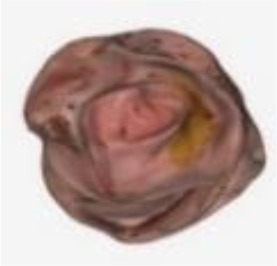	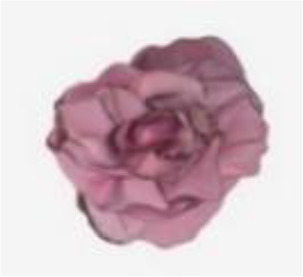
	82.47%		78.76%	75.29%	72.21%	69.66%	67.54%
RF-OOB-2		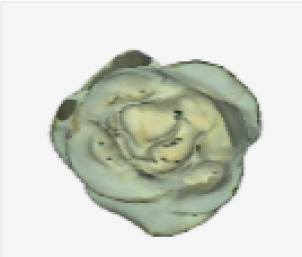	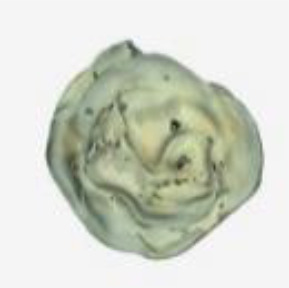	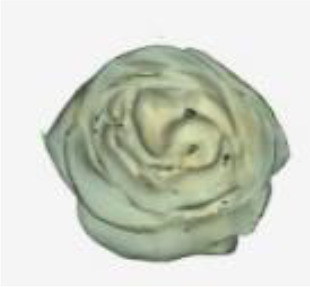	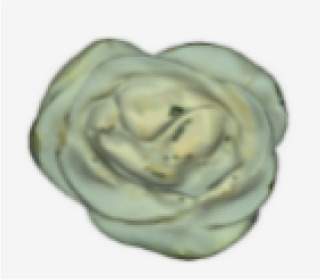	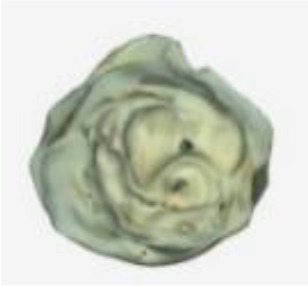	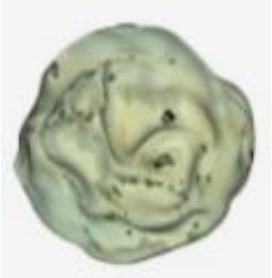
		88.72%	86.35%	83.44%	79.49%	76.17%	72.64%
RF-OOB-3		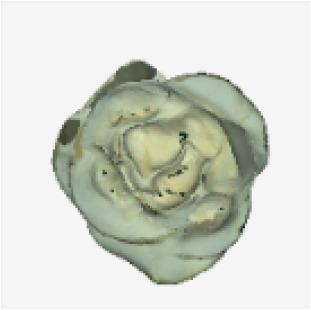	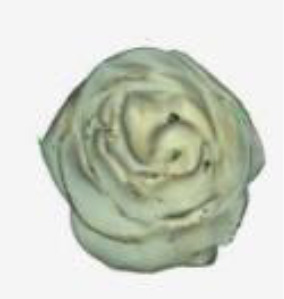	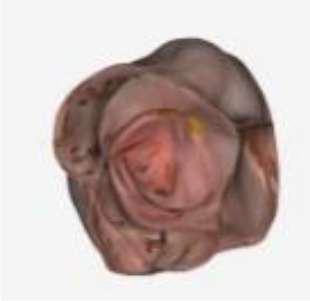	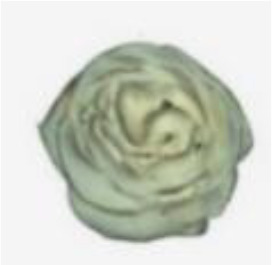	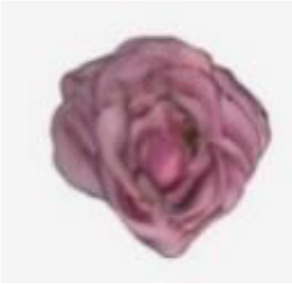	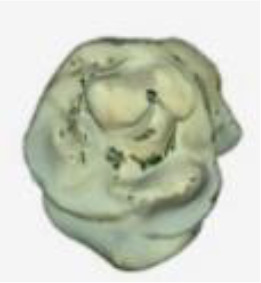
87.68%			85.13%	83.45%	78.05%	75.66%	71.72%

**Table 6:** Retrieval results of 3D model of Guerlain rose flowers.

Method	Retrieval similarity					
D2	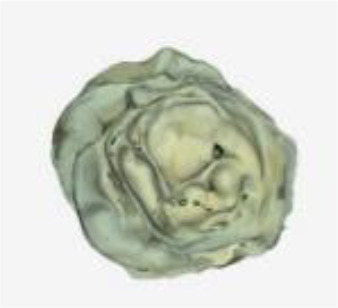	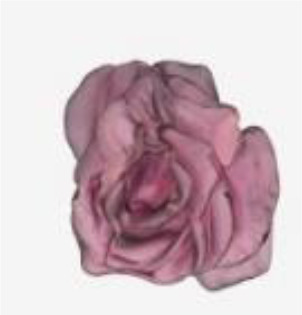	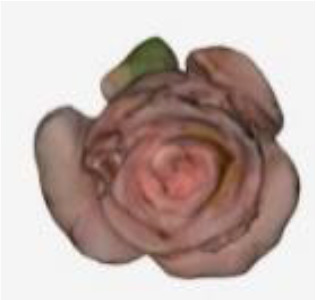	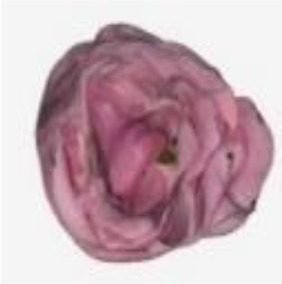	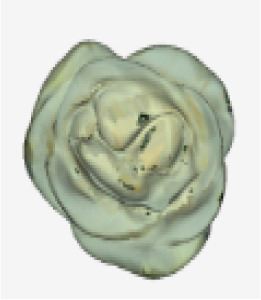	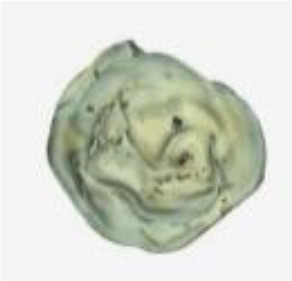
	81.93%	79.11%	75.97%	72.33%	69.18%	67.09%
RF-OOB-2	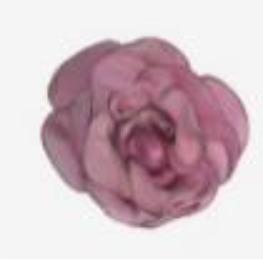	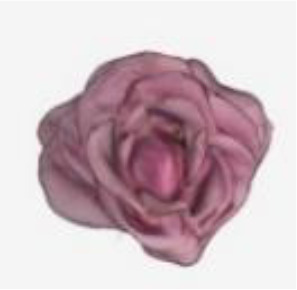	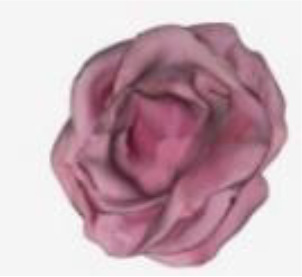	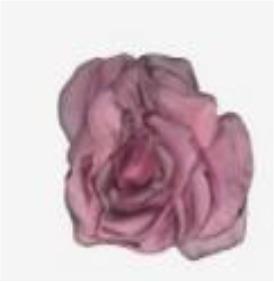	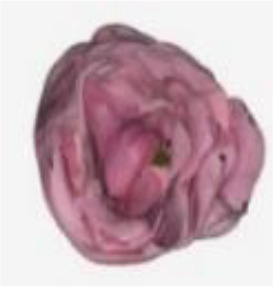	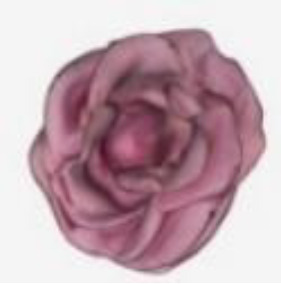
	87.48%	85.24%	81.67%	78.32%	74.16%	72.52%
RF-OOB-3	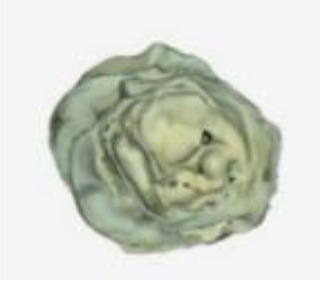	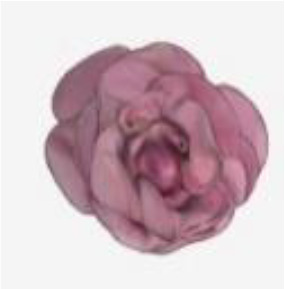	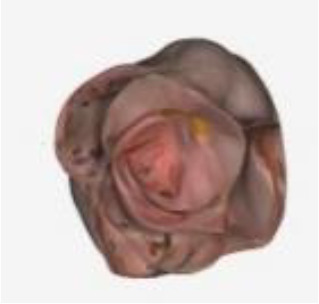	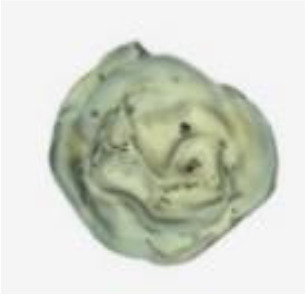	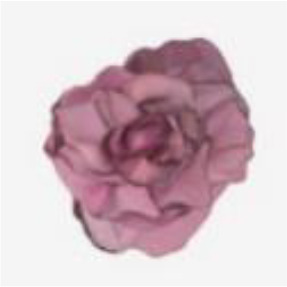	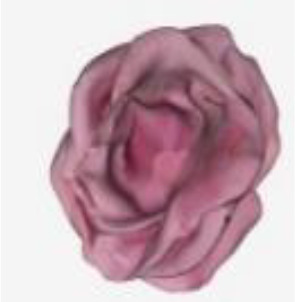
	84.96%	84.76%	81.79%	77.27%	73.95%	71.22%

According to the similarity with the input model, the models are arranged in sequence in **Tables 5**, **6**. Because the D2 shape-distribution algorithm is most commonly used in feature extraction and retrieval of 3D models, and the D2 shape-distribution method was applied as the control group, RF-OOB-2 and RF-OOB-3 were selected as the experimental groups. It can be seen from **Table 5** that, under the condition of ignoring color information, three different feature-extraction and retrieval methods can retrieve relevant models based on the input models, indicating that it is feasible to study the similarity retrieval of rose flowers in the 3D field. The model retrieved by RF-OOB-2 belongs to the same kind as the input model. In the output results of D2 and RF-OOB-3, it can be found the color of some retrieved models is inconsistent with the input model. The output model of D2 includes two orange and two pink models, and RF-OOB-3 outputs one orange and one pink model. This is because that the D2 and RF-OOB-3 methods do not contain color features. Although the flower shape features retrieved by D2 and RF-OOB-3 are very similar to the input model, the flower color in the retrieval results is inconsistent. Subjectively, it can be considered that the retrieval result of flower similarity is not good. It can be further perceived that while learning from the basic methods in the general field, it is necessary to consider the particularity of practical research objects. Color features are very necessary for 3D Chinese rose models. In terms of flower morphology, the further comparison reveals that results of RF-OOB-3 are better than those of D2, which means that the fused features can indeed improve the resolution of flower features and help optimize the retrieval results.

**Table 6** shows the similarity retrieval results with Guerlain roses as the input model. Compared with those presented in **Table 5**, the retrieval results of Guerlain and Swan antique rose flowers are basically similar, the retrieval result of RF-OOB-2 is the best, and the result of RF-OOB-3 is slightly better than that of D2. Based on the retrieval examples, RF-OOB-2 is a new method for Chinese roses’ feature extraction and retrieval that can meet the needs of conventional Chinese roses’ retrieval and classification.

## 4 Conclusion

This paper proposes a feature extraction and classification method based on 3D Chinese rose models, which can effectively classify different Chinese rose varieties. The sharpness and contour features combined with the color information were extracted from the 3D Chinese rose models. The RF-OOB method was adopted to rank the importance of the extracted features. A shape-feature descriptor based on the unique attributes of Chinese rose flowers was constructed. The experimental results prove that the sharpness and contour features are the typical features in 3D rose models, and the color information is an important reference feature to improve the classification accuracy. The fused features based on the exponential distribution weight method have an effective average classification accuracy of approximately 87%, which can meet the basic retrieval and classification needs of 3D Chinese rose models. In the future, the authors will continue optimizing and expanding the proposed retrieving and classification method for the other 3D flower models; and explore the application of AI and VR technology to the visualization and evaluation of 3D flower models.

## Data availability statement

The datasets presented in this article are not readily available because the dataset also forms part of an ongoing study. Requests to access the datasets should be directed to the corresponding author.

## Author contributions

All authors listed have made a substantial, direct and intellectual contribution to the work, and approved it for publication.

## Funding

This research was funded by the National Key R&D Plan Project (2018YFD1000400) and the Natural Science Foundation of Shandong Province (ZR2021QE217).

## Conflict of interest

The authors declare that the research was conducted in the absence of any commercial or financial relationships that could be construed as a potential conflict of interest.

## Publisher’s note

All claims expressed in this article are solely those of the authors and do not necessarily represent those of their affiliated organizations, or those of the publisher, the editors and the reviewers. Any product that may be evaluated in this article, or claim that may be made by its manufacturer, is not guaranteed or endorsed by the publisher.
